# A Goldilocks Principle for the Gut Microbiome: Taxonomic Resolution Matters for Microbiome-Based Classification of Colorectal Cancer

**DOI:** 10.1128/mbio.03161-21

**Published:** 2022-01-11

**Authors:** Courtney R. Armour, Begüm D. Topçuoğlu, Andrea Garretto, Patrick D. Schloss

**Affiliations:** a Department of Microbiology and Immunology, University of Michigan, Ann Arbor, Michigan, USA; University of Maryland School of Medicine

**Keywords:** 16S rRNA gene sequencing, colon cancer, machine learning, microbiome, taxonomic level

## Abstract

Colorectal cancer is a common and deadly disease in the United States accounting for over 50,000 deaths in 2020. This progressive disease is highly preventable with early detection and treatment, but many people do not comply with the recommended screening guidelines. The gut microbiome has emerged as a promising target for noninvasive detection of colorectal cancer. Most microbiome-based classification efforts utilize taxonomic abundance data from operational taxonomic units (OTUs) or amplicon sequence variants (ASVs) with the goal of increasing taxonomic resolution. However, it is unknown which taxonomic resolution is optimal for microbiome-based classification of colorectal cancer. To address this question, we used a reproducible machine learning framework to quantify classification performance of models based on data annotated to phylum, class, order, family, genus, OTU, and ASV levels. We found that model performance increased with increasing taxonomic resolution, up to the family level where performance was equal (*P* > 0.05) among family (mean area under the receiver operating characteristic curve [AUROC], 0.689), genus (mean AUROC, 0.690), and OTU (mean AUROC, 0.693) levels before decreasing at the ASV level (*P* < 0.05; mean AUROC, 0.676). These results demonstrate a trade-off between taxonomic resolution and prediction performance, where coarse taxonomic resolution (e.g., phylum) is not distinct enough, but fine resolution (e.g., ASV) is too individualized to accurately classify samples. Similar to the story of Goldilocks and the three bears (L. B. Cauley, *Goldilocks and the Three Bears*, 1981), mid-range resolution (i.e., family, genus, and OTU) is “just right” for optimal prediction of colorectal cancer from microbiome data.

## OBSERVATION

Colorectal cancer is one of the most common cancers in men and women and a leading cause of cancer-related deaths in the United States (1). Early detection and treatment are essential to increase survival rates, but for reasons such as invasiveness and high screening costs (i.e., colonoscopy), many people do not comply with recommended screening guidelines (2). This prompts a need for low-cost, noninvasive detection methods. A growing body of research points to the gut microbiome as a promising target for noninvasive detection of “screen relevant neoplasia” (SRN) consisting of advanced adenomas and carcinomas (3, 4). The diagnostic potential of the gut microbiome in detecting SRNs has been explored through machine learning (ML) methods using abundances of operational taxonomic unit (OTU) classifications based on amplicon sequencing of the 16S rRNA gene (3). Recent work has pushed for the use of amplicon sequence variants (ASVs) to replace OTUs for marker gene analysis because of the improved resolution with ASVs (5). However, it is unclear whether OTUs are the optimal taxonomic resolution for classifying SRNs from microbiome data or whether the additional resolution provided by ASVs is useful for ML classification. Topçuoğlu et al. (6) recently demonstrated how to effectively apply machine learning (ML) methods to microbiome-based classification problems and developed a framework for applying ML practices in a more reproducible way. This analysis utilizes the reproducible framework developed by Topçuoğlu et al. to determine which ML method and taxonomic level produce the best performing classifier for detecting SRNs from microbiome data.

**FIG 1:**
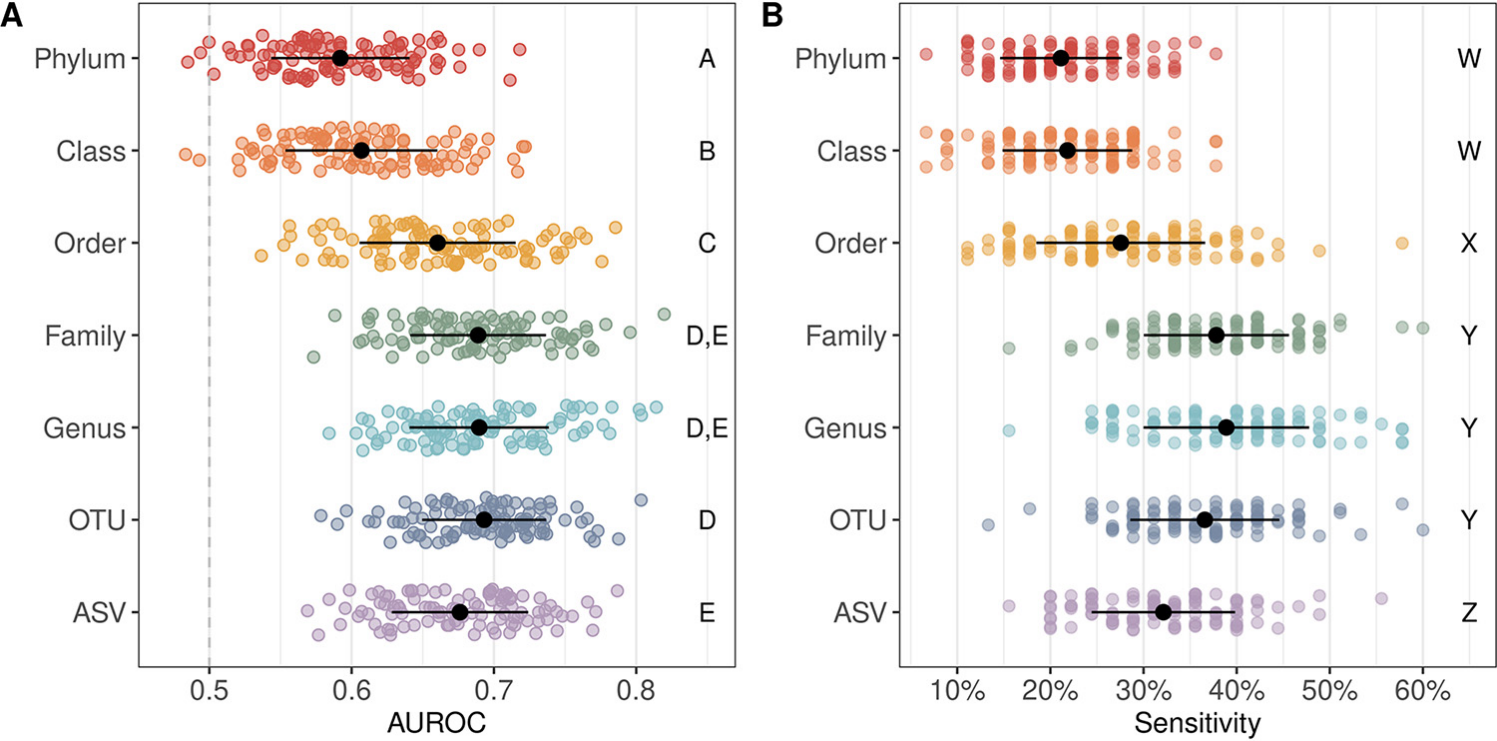
Random forest model performance. (A) Strip plot of the area under the receiver operating characteristic curve (AUROC) values on the test data set for 100 seeds predicting SRNs using a random forest model. Black circles denote the means, and black lines denote the standard deviations. The gray dashed line denotes an AUROC of 0.5 which is equivalent to random classification. Significance between taxonomic levels was quantified by comparing the difference in mean AUROC and is denoted by letters A through E on the right side of the plot; taxonomic levels with the same letter are in the same significance group and are not significantly different from one another. (B) Strip plot of the sensitivity at a specificity of 90% across the 100 model iterations for each taxonomic level. Black circles denote the means, and black lines denote the standard deviations. The letters W through Z on the right side of the plot denote the significance groups.

Utilizing publicly available 16S rRNA sequence data from the stools of patients with SRNs and healthy controls, we generated taxonomic abundance tables with mothur (7) annotated to phylum, class, order, family, genus, OTU, and ASV levels. Using the taxonomic abundance data and the mikropml R package (8), we quantified how reliably samples could be classified as SRN or normal using five machine learning methods, including random forest, L2-regularized logistic regression, decision tree, gradient boosted trees (XGBoost), and support vector machine with radial basis kernel (SVM radial). Across the five machine learning methods tested, model performance increased with increasing taxonomic level usually peaking around genus/OTU level before dropping off slightly with ASVs (see Fig. S1 in the supplemental material). Regardless of the taxonomic level, random forest (RF) models consistently had the largest area under the receiver operating characteristic curve (AUROC). Within the RF model, the highest AUROCs were observed for family (mean AUROC, 0.689), genus (mean AUROC, 0.690), and OTU (mean AUROC, 0.693) level data with no significant difference between the three (*P* > 0.05 [Fig. 1A and Fig. S2]). Performance with ASVs (mean AUROC, 0.676) was significantly lower than with OTUs (*P* < 0.01) but was comparable to family (*P* = 0.06) and genus (*P* = 0.05) levels (Fig. 1A). These results suggest that increased resolution improves model performance up to the OTU level where further taxonomic resolution is not necessarily better for identifying individuals with SRNs based on microbiome composition.

10.1128/mBio.03161-21.1 FIG S1 Model performance across taxonomy. Boxplots of AUROC values from predicting whether samples came from subjects with screen relevant neoplasias (i.e., advanced adenoma or cancer) or healthy controls across five machine learning methods, including random forest, L2-regularized logistic regression (logistic regression), decision tree, gradient boosted trees (XGBoost), and support vector machine with radial basis kernel (SVM radial). Due to the random split of data into training and testing sets, each model was run across 100 seeds to account for variation in training/test data splits. Download FIG S1, TIF file, 0.2 MB. Copyright © 2022 Armour et al. 2022 Armour et al. https://creativecommons.org/licenses/by/4.0/ This content is distributed under the terms of the Creative Commons Attribution 4.0 International license.

10.1128/mBio.03161-21.2 FIG S2 Averaged ROC curves. ROC curves with averaged true-positive rate (or sensitivity) across the 100 iterations of the random forest model. The shaded region represents the standard deviations from the means. The dashed line represents an AUROC of 0.5, which is equivalent to random classification. The mean AUROC for each taxonomic level is printed on the bottom right of the plot. Download FIG S2, TIF file, 0.2 MB. Copyright © 2022 Armour et al. 2022 Armour et al. https://creativecommons.org/licenses/by/4.0/ This content is distributed under the terms of the Creative Commons Attribution 4.0 International license.

While comparing AUROC values between models is a useful way to assess the overall model performance, AUROC values summarize the performance across all thresholds and can be misleading since models with the same AUROC can have different ROC curve shapes (9). Depending on the intended implementation of the model, one may want to optimize sensitivity over specificity or vice versa. In this case, the optimal model will detect as many true-positive results (people with SRNs) as possible. To further compare the model performance across taxonomic levels, we compared the sensitivity of the models at a specificity of 90%. The highest sensitivity values were observed for family (mean sensitivity, 0.38), genus (mean sensitivity, 0.39), and OTU (mean sensitivity, 0.37) level data (*P* > 0.05 [Fig. 1B]), consistent with the AUROC results. Phylum (mean sensitivity, 0.21), class (mean sensitivity, 0.22), order (mean sensitivity, 0.28), and ASV (mean sensitivity, 0.32) sensitivity values were all significantly lower than family, genus, and OTU sensitivity values (*P* < 0.05 [Fig. 1B]). This analysis further supports the observation that finer resolution does not improve SRN detection.

One hypothesis for the observation that model performance increases from phylum to OTU level then drops at the ASV level is that at higher taxonomic levels (e.g., phylum), there are too few taxa and too much overlap to reliably differentiate between cases and controls. At the level of genus or OTU, there is enough data and variation, but at the ASV level, the data are too specific to individuals and do not overlap enough. Examination of the prevalence of taxa in samples at each level supports this idea. A majority of taxa were present in greater than 70% of samples at the phylum (67% of taxa) and class (63% of taxa) levels. The opposite was observed at the OTU and ASV levels where 50% and 41% of taxa, respectively, were present in only 20% or less of the samples (Fig. S3). Of note, the ML pipeline includes a preprocessing step that occurred prior to training and classifying the ML models. For example, perfectly correlated taxa provide the same information to build the model and thus can be collapsed. Additionally, features with zero or near-zero variance across samples were removed. Interestingly, despite starting with 104,106 ASVs, only 478 (0.5%) remained after preprocessing. At the OTU level, 705 of the 20,079 OTUs (3.5%) remained after preprocessing (Table 1). While the resolution provided by ASVs is useful in certain contexts (10, 11), these results suggest that the resolution is too fine for use in machine learning classification of SRNs based on microbiome composition.

**TABLE 1:** Overview of the number of features at each taxonomic level before and after preprocessing as described in Materials and Methods

Taxonomic level	No. of features		% of features kept after preprocessing
	Before preprocessing	After preprocessing	
Phylum	19	9	47.4
Class	36	19	52.8
Order	65	28	43.1
Family	124	54	43.5
Genus	316	115	36.4
OTU	20,079	705	3.5
ASV	104,106	478	0.5

10.1128/mBio.03161-21.3 FIG S3 Prevalence of taxa in samples. Distribution of the prevalence of taxa across samples at each taxonomic level. The percentage of samples is split into 10 groups where the first is for taxa present in 0 to 10% of samples, then >10% to 20% of samples, and so on. The total number of taxa for each taxonomic level after preprocessing is in parentheses next to the title of the plot. Download FIG S3, TIF file, 0.2 MB. Copyright © 2022 Armour et al. 2022 Armour et al. https://creativecommons.org/licenses/by/4.0/ This content is distributed under the terms of the Creative Commons Attribution 4.0 International license.

A look into the most important taxa at each level for classifying samples revealed some nesting where several genera and their higher taxonomic classifications were in the top 10 most important taxa (Fig. S4). For example, the genus *Gemella* was an important taxon at the genus and OTU levels, and its higher classifications were also important (*Firmicutes* > *Bacilli* > *Bacillales* > *Bacillales* Incertae Sedis XI > *Gemella*). *Fusobacterium* displayed a similar pattern, except that the family level classification (*Fusobacteriaceae*) importance was ranked 16th out of 54 families. In the case of unclassified *Lachnospiraceae*, there were several OTUs with this label that were in the top 10; however, at the genus level, this taxon was ranked lower in importance (21st out of 115 genera), suggesting there may be some benefit to separating different taxonomic groupings within *Lachnospiraceae*.

10.1128/mBio.03161-21.4 FIG S4 Top 10 important taxa at each taxonomic level. Summary of the 10 most important taxa for the random forest models at each taxonomic level based on the average decrease in AUROC when the feature is permuted. Each circle represents the mean decrease in AUROC, and the lines extending from the circle represent the standard deviations from the mean. Taxonomic annotations were assigned only to the genus level; therefore, the OTU and ASV plots have genus labels. Download FIG S4, TIF file, 0.8 MB. Copyright © 2022 Armour et al. 2022 Armour et al. https://creativecommons.org/licenses/by/4.0/ This content is distributed under the terms of the Creative Commons Attribution 4.0 International license.

These results demonstrate a Goldilocks effect (12) such that consideration of the appropriate taxonomic resolution for utilizing the microbiome as a predictive tool is warranted. In general, we found that finer taxonomic resolution (e.g., ASV) did not add additional sensitivity to predicting SRNs based on microbiome composition. Family, genus, and OTU level data all performed similarly. At the ASV level, the fine resolution actually impeded model performance due to the sparsity of shared taxa and led to decreased model performance. The tendency for ASV level annotation to split single bacterial genomes into multiple taxa (13) could also be a contributing factor to the sparsity of shared taxa. Additionally, these results indicate that there are not specific individual bacterial strains that are useful to resolve SRNs, rather sets of closely related bacterial taxa. Overall, either family, genus, or OTU level taxonomy appear to perform similarly for predicting subjects with SRNs based on the composition of the gut microbiome. A potential benefit of utilizing genus or family level data could be that it may allow for merging data generated from different 16S rRNA gene regions or sequencing platforms. Although this analysis focused on a single disease and data set from that disease, we suspect that because of the patchy nature of the human microbiome the general observation from our analysis will hold in other diseases. Rather than interrogating data at the finest possible scale, we encourage researchers to explore this concept further by identifying the coarsest taxonomic level that provides the greatest signal between groups.

### Data set.

Raw 16S rRNA gene amplicon sequence data isolated from human gut samples (14) was downloaded from NCBI Sequence Read Archive (accession no. SRP062005). This data set contains stool samples from 490 subjects. Based on the available metadata, samples categorized as normal, high risk normal, or adenoma were labeled “normal” for this analysis, and samples categorized as advanced adenoma or carcinoma were labeled as “screen relevant neoplasia” (SRN). This resulted in a total of 261 “normal” samples and 229 “SRN” samples.

### Data processing.

Sequence data were processed with mothur (1.44.3) (7) using the SILVA reference database (v132) (15) to produce count tables for phylum, class, order, family, genus, OTU, and ASV following the Schloss Lab MiSeq standard operating procedure (SOP) described on the mothur website (https://mothur.org/wiki/miseq_sop/). ASV level data were also produced using DADA2 (16) to ensure consistent results with a different pipeline. Data were processed following the DADA2 pipeline, but setting pool=TRUE to infer ASVs from the whole data set rather than per sample. The resulting ASV table was subsampled for consistency with the mothur data. The DADA2-generated ASVs performed worse than the mothur-generated ASVs (DADA2 ASV mean AUROC, 0.659; *P* < 0.05).

### Machine learning.

Machine learning models were run with the R package mikropml (v0.0.2) (8) to predict the diagnosis category (normal versus SRN) of each sample. Data were preprocessed to normalize values (scale/center), remove values with zero or near-zero variance, and collapse colinear features using default parameters. Initially, the models were run with default hyperparameters, but the models were expanded if the peak performance was at the edge of the hyperparameter range. Each taxonomic model taxonomic level combination (e.g., random forest on genus) was run with 100 different seeds. Each seed split the data into a training (80%) and testing (20%) set, and output performance of the training and testing as area under the receiver operating curve (AUROC).

To compare performance between taxonomic levels and models, *P* values were calculated as previously described (6). To compare sensitivity at 90% specificity, probabilities on the test data set were collected for each seed and used to calculate sensitivity for specificity values ranging from 0 to 1 in 0.01 increments. The sensitivity at a specificity of 90% was pulled for each seed. The averaged ROC curves were plotted by taking the average and standard deviation of the sensitivity at each specificity value. An optional output from the mikropml package is the permuted feature importance which is quantified by iteratively permuting each feature in the model and assessing the change in model performance. Features are presumed to be important if the performance of the model, measured by the AUROC, decreases when that feature is permuted. Ranking of feature importance was determined by ordering the features based on the average change in AUROC across the 100 seeds where features with a larger decrease in AUROC are ranked higher in importance.

To quantify prevalence of the features, the number of samples with nonzero abundance was divided by the total number of samples resulting in values ranging from 0 to 1 where 0 indicates the feature is not found in any samples, 0.5 indicates the feature is found in half of the samples, and 1 indicates the feature is found in all of the samples.

### Data availability.

All code is available at https://github.com/SchlossLab/Armour_Resolution_mBio_2021.
