# Supplementary figures and images for: A Goldilocks Principle for the Gut Microbiome: Taxonomic Resolution Matters for Microbiome-Based Classification of Colorectal Cancer
Source: mBio. 2022 Jan 11;13(1):e03161-21. doi: 10.1128/mbio.03161-21 (PMC8749421; doi:10.1128/mbio.03161-21)

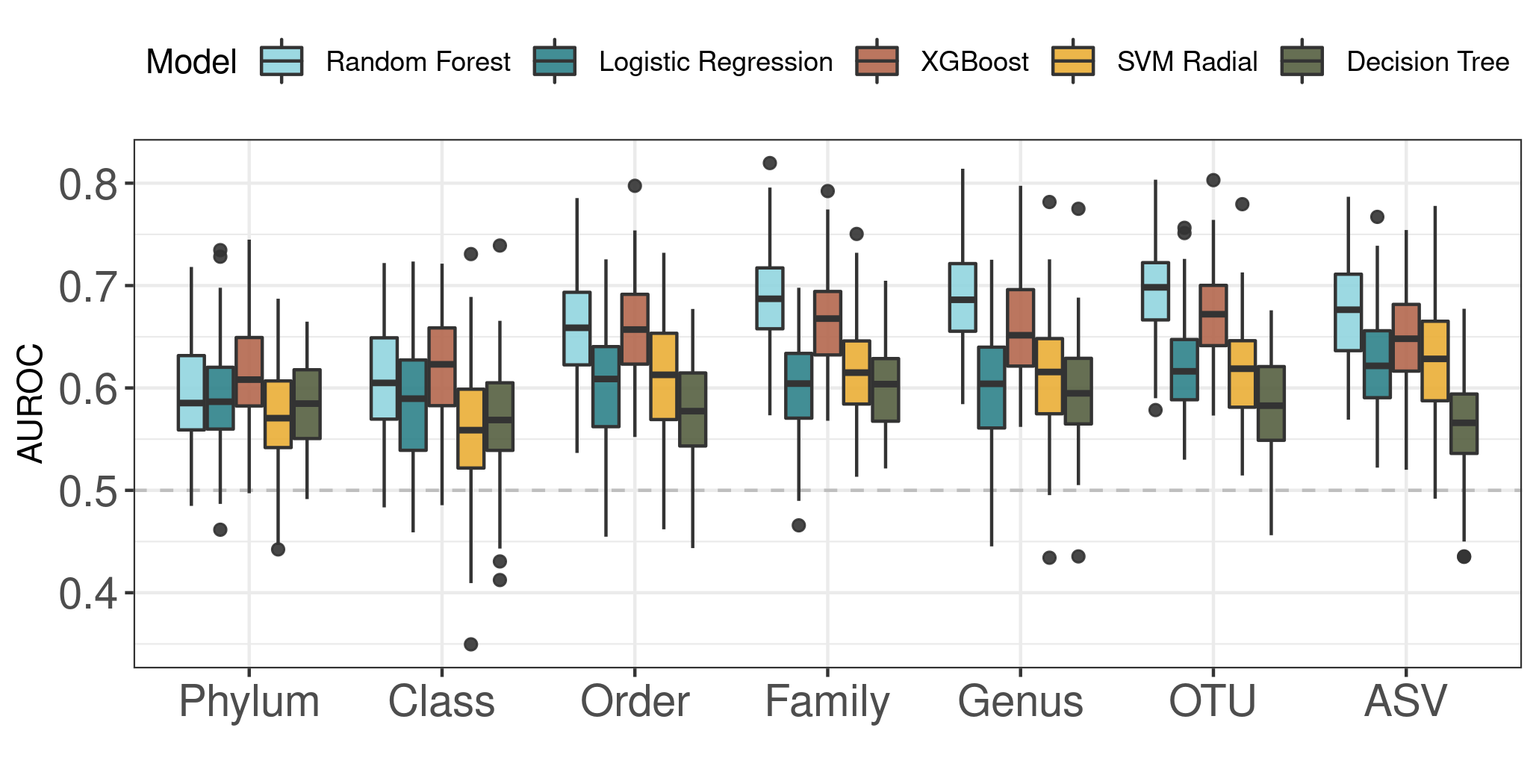

Supplement: FIG S1 [file mbio.03161-21-sf001.tif]

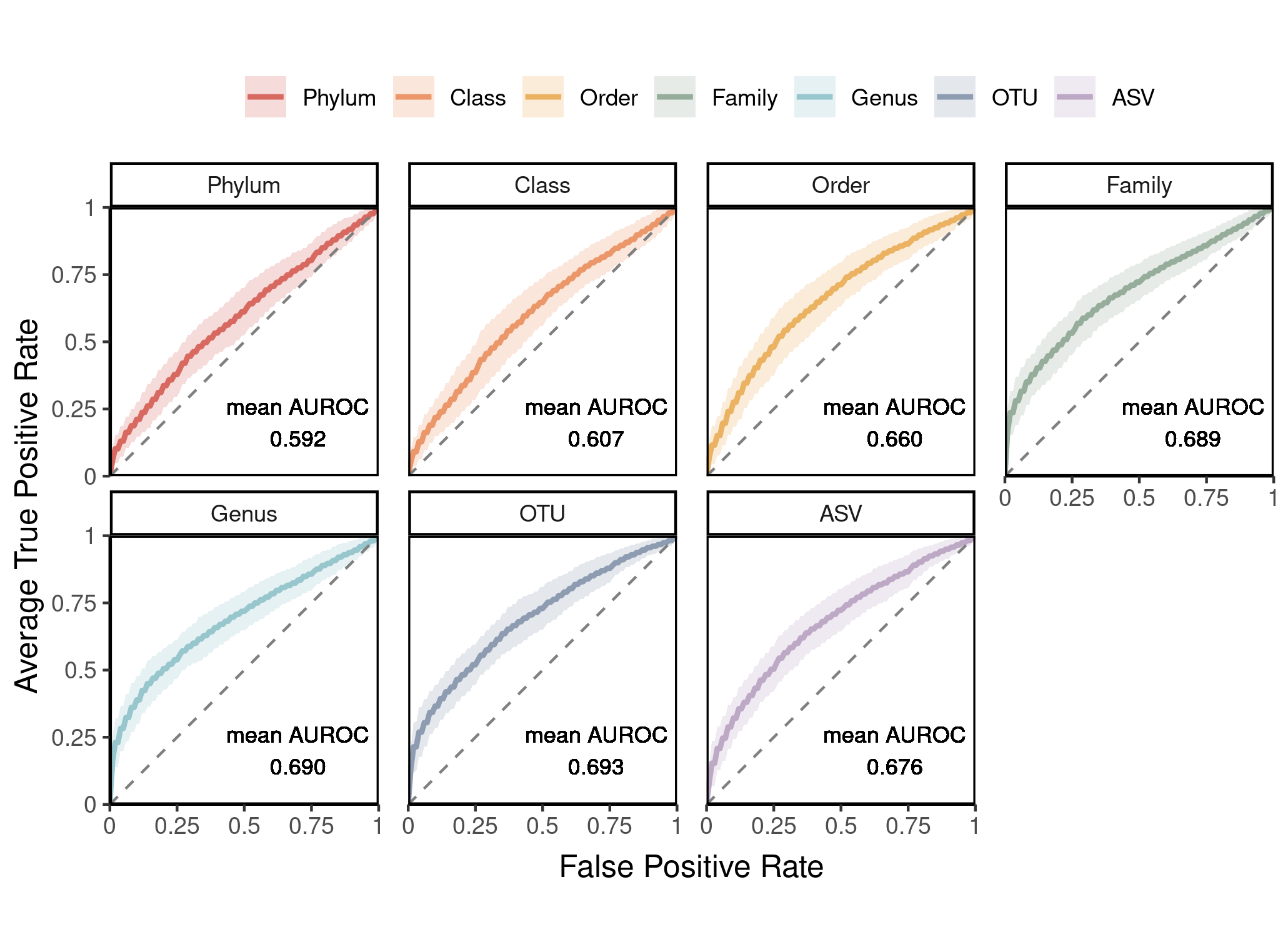

Supplement: FIG S2 [file mbio.03161-21-sf002.tif]

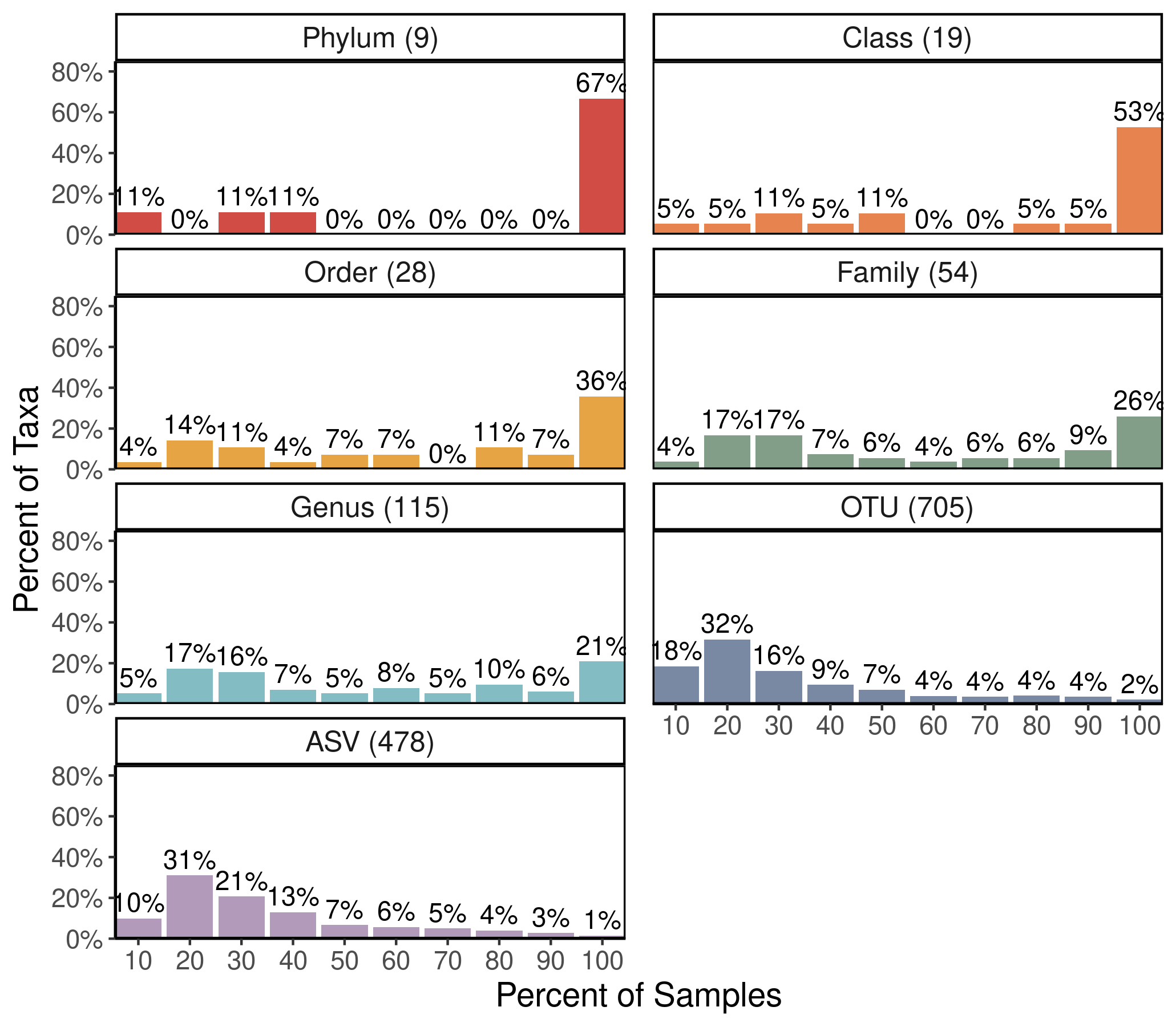

Supplement: FIG S3 [file mbio.03161-21-sf003.tif]

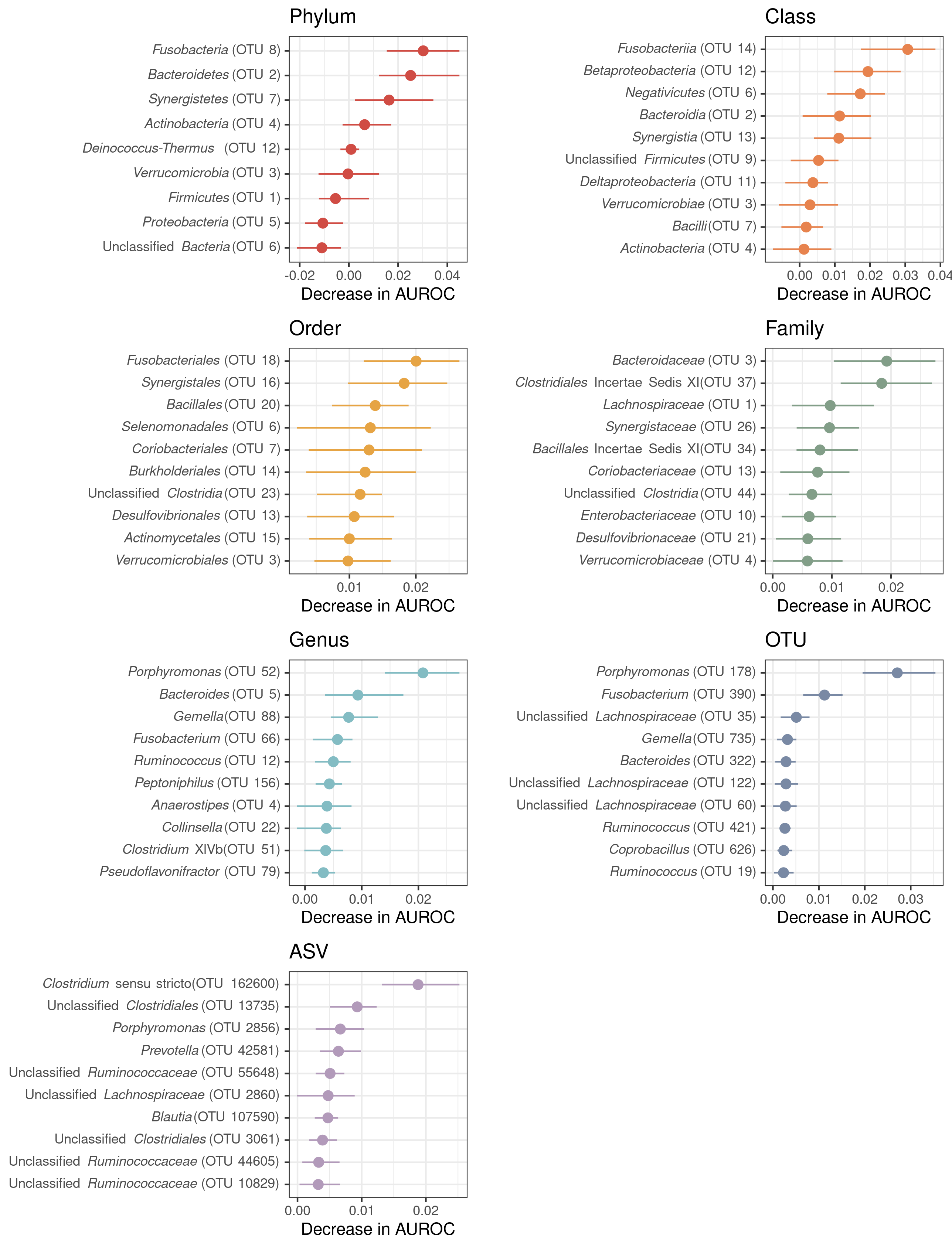

Supplement: FIG S4 [file mbio.03161-21-sf004.tif]
